# Public Knowledge, Attitudes, and Preventive Practices Toward G6PD Deficiency in Al-Kharj, Saudi Arabia: A Community-Based Cross-Sectional Study

**DOI:** 10.3390/healthcare13243261

**Published:** 2025-12-12

**Authors:** Noura Al-Dayan

**Affiliations:** Department of Medical Laboratory, College of Applied Medical Sciences, Prince Sattam bin Abdulaziz University, Al-Kharj 16278, Saudi Arabia; n.aldayan@psau.edu.sa

**Keywords:** G6PD deficiency, public awareness, preventive practices, cross-sectional study, genetic screening, Saudi Arabia, health promotion

## Abstract

**Highlights:**

**What are the main findings?**

- Knowledge of G6PD deficiency in Al-Kharj was limited, with major gaps in inheritance, medication triggers, and non-fava dietary risks.
- Preventive behaviours were low, with minimal uptake of premarital counselling and genetic testing.

**What are the implications of the main findings?**

- Targeted education across premarital, antenatal, and primary-care services is needed to reduce preventable haemolysis.
- Embedding G6PD awareness in routine care supports Vision 2030 goals for preventive health and improved genetic counselling pathways.

**Abstract:**

Background: Glucose-6-phosphate dehydrogenase (G6PD) deficiency is the world’s most prevalent X-linked enzymopathy, yet public literacy regarding its inheritance, haemolytic triggers, and preventive actions remains inadequate in many high-risk populations. This study assessed public knowledge, attitudes, and preventive practices toward G6PD deficiency among adults in Al-Kharj, Saudi Arabia, a region reporting haemoglobinopathy burden and a recent expansion of national newborn screening. Materials and Methods: A community-based cross-sectional survey was administered between May and September 2025 using a bilingual, self-administered questionnaire. A total of 1104 adults (≥18 years) were recruited through convenience and snowball sampling. Knowledge was scored using 13 dichotomous factual items, and findings are reported as proportions with corresponding 95% confidence intervals. Results: Participants were predominantly female (57%) and university-educated (34.2%). Although 58.5% had heard of “fava bean anaemia”, only 38% recognised the X-linked mode of inheritance and 36.1% identified medication-induced haemolysis, despite 61.8% correctly linking fava beans to haemolytic risk. The mean knowledge score across items was 34.4%. Preventive practices were limited: 41.5% reported premarital medical consultation, and only 21.6% had undergone genetic assessment. Conclusions: Despite national advances in newborn screening, substantial public knowledge deficits and low engagement with preventive practices persist. Strengthened community-level education, particularly regarding inheritance, medication safety, and proactive screening, may reduce preventable haemolysis events. Integrating G6PD-targeted messaging within premarital, antenatal, and primary-care services may support long-term preventive health objectives.

## 1. Introduction

Glucose-6-phosphate dehydrogenase (G6PD) deficiency is the most common inherited enzymopathy worldwide, affecting approximately 400 million people [1,2]. The prevalence is highest in tropical and subtropical regions, particularly in Africa, Asia, the Middle East, and the Mediterranean [3,4]. G6PD deficiency is an X-linked disorder caused by pathogenic variants in the G6PD gene, leading to variable enzyme activity levels [5]. G6PD is the rate-limiting enzyme of the oxidative pentose–phosphate pathway, generating nicotinamide adenine dinucleotide phosphate (NADPH), which protects red blood cells from oxidative injury [6].

Clinically, the disorder manifests across a broad spectrum—from asymptomatic cases to acute haemolytic anaemia precipitated by infections, contraindicated drugs, or the ingestion of fava beans [7,8]. In Saudi Arabia, the burden of G6PD deficiency remains high, with marked regional heterogeneity. The Eastern Province has the highest male frequency in Saudi Arabia [9]. Although neonatal screening for G6PD deficiency was incorporated into the national newborn screening panel in 2023, adult testing is not yet routine within premarital screening programmes [9,10]. Consequently, many individuals remain unaware of their carrier status until a haemolytic episode occurs [11]. Knowledge and health-seeking behaviour toward G6PD deficiency are critical determinants of early diagnosis, avoidance of oxidative triggers, and timely medical consultation. Several Saudi studies have examined the parental awareness of neonatal G6PD deficiency [12,13]. Adult community knowledge remains underexplored, particularly in central regions such as Al-Kharj. This study therefore assessed the knowledge, attitudes, and practices (KAP) toward G6PD deficiency among adults in Al-Kharj, Saudi Arabia. Understanding public awareness and behavioural patterns can inform community-based prevention, clinical counselling, and educational interventions that support national goals for genetic disease prevention under Vision 2030.

## 2. Materials and Methods

### 2.1. Study Design and Setting

This descriptive, cross-sectional study assessed the knowledge, attitudes, and practices (KAP) regarding glucose-6-phosphate dehydrogenase (G6PD) deficiency among residents of Al-Kharj, Saudi Arabia. The study was conducted between May and September 2025. Eligible participants were Saudi adults aged 18 years or older residing in Al-Kharj. Inclusion required the ability to read Arabic or English and the willingness to provide informed consent.

Convenience and snowball sampling were employed due to the absence of a community sampling frame in Al-Kharj. Online recruitment represents a practical and widely used approach for reaching diverse adults in community-based KAP studies, particularly when probability sampling is not feasible. The limitations of this approach, including potential selection bias, are acknowledged in the Discussion.

A cross-sectional design was selected as it is well suited for assessing population-level knowledge, attitudes, and practices in public-health research and has been widely used in similar studies of hereditary and genetic conditions. The methodological steps were reported according to recommended standards for cross-sectional KAP studies to ensure clarity, transparency, and reproducibility.

### 2.2. Study Population and Sampling

Participants were recruited through convenience sampling using online snowballing via social media platforms and QR code dissemination, supplemented by limited in-person distribution. A total of 1104 participants completed the survey. Although no formal power calculation was performed, the achieved sample size substantially exceeded the minimum recommended for cross-sectional KAP surveys. Based on an estimated adult population of approximately 400,000 in Al-Kharj, this sample provided adequate representation to estimate proportions with 95% confidence and a margin of error of less than 5%, assuming moderate response variability. 

### 2.3. Data Collection Instrument

Data were collected using an anonymous self-administered questionnaire distributed primarily online. The bilingual instrument comprised 35 items, including three sociodemographic questions (age group, gender, and education level) and 32 items assessing knowledge, attitudes, and practices. The knowledge domain included 13 factual items covering (1) inheritance pattern (X-linked), (2) drug-related haemolysis, (3) symptoms such as pallor, jaundice, and dyspnoea, and (4) dietary triggers including fava beans, falafel made with fava beans, and non-fava legumes such as chickpeas, lentils, and berries.

The questionnaire was developed by adapting items from previously validated KAP instruments addressing G6PD deficiency and hereditary genetic conditions, with additional context-specific items added after expert consultation. Content validity was assessed by two specialists (a medical geneticist and a public health expert), who independently evaluated each item for relevance, clarity, and cultural appropriateness. A pilot test with 15 adults was conducted to confirm comprehension, clarity, and logical flow. Internal consistency reliability was acceptable for population-level KAP assessment (knowledge α = 0.78; attitudes α = 0.81; practices α = 0.76). The complete questionnaire is provided in Supplementary File S1.

Each correct answer was scored as 1, and incorrect or “don’t know/not applicable” responses were scored as 0. This binary approach is widely used in KAP research to ensure conservative and consistent interpretation. Total knowledge scores were calculated by summing correct responses, and a ≥50% threshold was used to define adequate knowledge. This midpoint cut-off is commonly applied in KAP studies when validated scoring benchmarks are unavailable and has been used in regional investigations of hereditary conditions and public-health awareness [14,15,16,17]. The aggregate knowledge score represented the proportion of correct responses across items. Confidence intervals (95% CIs) for key proportions were calculated as described in the Statistical Analysis section.

### 2.4. Statistical Analysis

The data were analysed using descriptive statistics, including frequencies and percentages. The data were analysed via GraphPad Prism, version 10.4.2 (GraphPad Software, LLC, San Diego, CA, USA) and Microsoft Excel 2013 (Microsoft Corporation, Redmond, WA, USA). Categorical variables are summarised as counts and percentages with corresponding 95% confidence intervals (CIs) for key proportions calculated using the Wilson method. Content validity was established via expert review.

Inferential statistical tests (e.g., chi-square analysis) and regression modelling were not conducted because the non-probability sampling design and subgroup structure did not provide sufficient statistical power for valid inferential or multivariable analyses. Performing such tests could yield unstable or misleading estimates; therefore, the analysis was restricted to descriptive statistics to maintain methodological rigour. ‘Don’t know’ responses were treated as valid response categories rather than missing data, and no missing responses occurred because the online survey required completion of all items.

## 3. Results

### 3.1. Sociodemographic Characteristics

A total of 1104 participants completed the survey. The majority were female (57.0%, *n* = 629), whereas 43.0% (*n* = 475) were male. The participants were categorised into five age groups; the most represented group was 28–37 years (35.5%, *n* = 392), whereas the 48–57-year group was the least represented (6.0%, *n* = 66). With respect to education, 34.2% (*n* = 378) held a university degree, whereas 15.9% (*n* = 176) had secondary-level education (Table 1).

### 3.2. General Awareness of G6PD Deficiency

General awareness of G6PD deficiency varied across participants (Table 2). More than half had heard of “fava-bean anaemia,” and approximately one-half recognised G6PD deficiency as an inherited disorder. However, awareness of the X-linked inheritance pattern was notably lower, with fewer than 40% identifying it correctly and over 40% reporting uncertainty. Family history awareness was also limited; although one-quarter reported a positive family history, almost two-thirds were unsure. These patterns suggest that while basic familiarity with the condition is relatively common, deeper genetic understanding remains limited across the community.

### 3.3. Knowledge of Symptoms

Awareness of key clinical symptoms associated with G6PD-related haemolysis varied considerably across the sample (Table 3). Jaundice was the most recognised symptom, with 54.2% of participants identifying it correctly, although 38.4% remained uncertain. In contrast, awareness of pallor—another classical sign of haemolysis—was relatively low (36.3%), with a similar proportion responding “Don’t know” (38.8%).

Knowledge of non-specific or early symptoms showed even greater uncertainty. More than half of respondents (57.2%) were unsure whether symptoms such as loss of appetite, nausea, diarrhoea, or vomiting were related to G6PD deficiency, and only 35.2% identified them as possible indicators.

Similarly, only about one-third of participants recognised shortness of breath (35.1%) and severe outcomes such as death or physical complications (34.4%) as potential consequences of acute haemolytic episodes, while approximately 39–40% were uncertain across these items.

Overall, these findings indicate that participants were more familiar with visible, well-known signs such as jaundice, whereas early warning symptoms and serious complications were poorly understood, reflecting a substantial gap in symptom-related knowledge.

### 3.4. Knowledge of Triggers and Preventive Practices

Knowledge of dietary and medication triggers varied substantially across participants (Table 4). More than half correctly recognised fava beans as a major trigger of haemolysis (56.6%), although 37.8% remained uncertain. Misconceptions were common for other legumes: over half of the respondents believed that hummus/chickpeas (51.2%) and lentils (51.1%) could trigger haemolysis, despite these foods not being established triggers of G6PD-related haemolysis. A similar misconception was seen for falafel, possibly due to its common association with fava beans.

Participants also demonstrated considerable uncertainty regarding less frequently discussed foods. For example, 41.0% responded “Don’t know” when asked about blueberries, and only 26.2% correctly identified them as non-triggers. Awareness of medication-related haemolysis was similarly limited. Only about one-third recognised aspirin, antimalarial drugs, or sulphonamide antibiotics as potential triggers, and more than 40% selected “Don’t know” for each item. Several medication-related triggers identified by participants were incorrect, highlighting confusion in this domain.

Overall, “Don’t know” responses ranged from 38% to 58% across the trigger-related items. These findings reflect substantial confusion between true G6PD triggers and unrelated foods or medications, underscoring the need for targeted public-health education to address persistent misconceptions.

### 3.5. Overall Knowledge and Practices

Overall knowledge was low, with participants answering correctly only about one-third of the items on the 13-item knowledge scale (34.4%; 95% CI 33.6–35.1). Recognition of the X-linked inheritance pattern remained limited (38.0%), and correct identification of dietary and medication triggers varied widely. While most participants correctly identified fava beans as a trigger, accuracy was much lower for medication-related items, reflecting substantial uncertainty in this domain.

Preventive practices were similarly modest. Fewer than half of the participants reported having attended premarital medical consultation, and only one-fifth had ever undergone genetic assessment. Postnatal confirmation of G6PD status was also limited, and many respondents were unsure whether they would seek consultation. These findings indicate gaps not only in knowledge but also in engagement with preventive health services. Full details are provided in Table 5.

### 3.6. Attitudes Toward G6PD Deficiency

Attitudes showed mixed levels of understanding among participants (Table 6). Nearly half viewed G6PD deficiency as a serious public health issue and agreed that affected individuals require lifelong monitoring. Awareness of genetic risk was somewhat higher, with more than half recognising consanguineous marriage as a contributing factor. In contrast, attitudes toward reproductive decisions were highly uncertain: only a minority supported avoiding pregnancy in families with an affected child, and more than half selected “Don’t know.” Overall, uncertainty was common across all attitude items, indicating limited confidence in interpreting the broader implications of the condition.

## 4. Discussion

Glucose-6-phosphate dehydrogenase (G6PD) deficiency is highly prevalent across the Mediterranean and Middle East, including Saudi Arabia [13]. National estimates vary widely (0–52%) due to regional heterogeneity in ancestry, screening coverage, and diagnostic practices [14]. Previous Saudi studies—mostly focused on neonates or affected families—have reported moderate awareness [15,16], and community-based surveys from the Eastern Province and Jazan have described “fair” knowledge (~50–53.6%) [17]. To our knowledge, this is the first study to assess community-level knowledge, attitudes, and practices (KAP) regarding G6PD deficiency in Al-Kharj.

In this large community sample, overall knowledge was limited (34.4%; 95% CI 33.6–35.1), lower than estimates reported in some central-region studies [18]. These differences may reflect local variability in health-education efforts, counselling access, and engagement with genetic screening programmes. Item-level findings highlight substantial gaps: although respondents frequently recognised fava beans as a trigger and jaundice as a key symptom, fewer correctly identified X-linked inheritance, medication-related haemolysis (e.g., antimalarials, sulphonamides), or distinguished non-fava foods such as chickpeas and lentils from true triggers. Preventive practices were also modest, with only 41.5% reporting premarital consultation and 21.6% having undergone genetic assessment.

Findings from Al-Kharj align with patterns reported in other regions of Saudi Arabia. Studies from Jazan, the Eastern Province, and Riyadh similarly observed moderate general awareness alongside persistent misconceptions about dietary triggers and medication-induced haemolysis [18,19,20]. A notable feature across all studies, including the present one, is the high proportion of “Don’t know” responses, especially for medication-related items [21,22,23]. This may reflect limited pharmacogenetic counselling in routine care, low visibility of drug–disease interaction education within pharmacy settings, and broader gaps in health literacy.

International comparisons show similar trends. Studies from Turkey, Oman, Jordan, and the United Arab Emirates have reported comparable misunderstanding of medication triggers and limited recognition of X-linked inheritance [21,22,23]. These parallels suggest that G6PD-related knowledge gaps represent a regional Middle Eastern public-health challenge rather than a locality-specific issue. Such consistency underscores the need for coordinated, culturally aligned education strategies across the region.

Several contextual factors may explain why misconceptions persist. In Middle Eastern dietary culture, legumes such as chickpeas, lentils, and ful are often grouped together, which may lead individuals to assume they share similar haemolytic potential. This cultural clustering may contribute to the widespread belief that non-fava legumes are unsafe for individuals with G6PD deficiency. Reliance on informal information sources—including family advice, social media, and community narratives—may reinforce inaccurate perceptions. Limited pharmacogenetic counselling and low awareness about medication-induced haemolysis may further contribute to uncertainty, particularly regarding aspirin, antimalarials, and sulphonamides. These contextual and cultural influences likely shape public understanding and help explain the high levels of uncertainty observed in this study.

These findings are especially relevant for Al-Kharj, where the burden of haemoglobinopathies remains notable [24]. Practical, low-cost interventions could include (i) clinic- and pharmacy-based trigger lists with brief counselling prompts; (ii) electronic medical record alerts to flag contraindicated medications for at-risk individuals; and (iii) public-facing education embedded within newborn screening programme and premarital services. Such measures align with Saudi Vision 2030 priorities to improve preventive health literacy, strengthen family counselling pathways, and reduce avoidable haemolytic crises.

### 4.1. Strengths and Limitations

This study benefits from a large community-based sample and detailed item-level analysis across general knowledge, symptom recognition, and dietary/medication-trigger domains. The comprehensive coverage of key constructs allowed for identification of specific knowledge gaps relevant to preventive health and counselling pathways in Al-Kharj.

However, several limitations must be acknowledged. The use of convenience sampling introduces potential selection bias and restricts generalizability. Online data collection may underrepresent older adults and individuals with lower digital literacy, biassing the sample toward younger, digitally active populations. Because aggregated data were used at the analysis stage and the study relied on a non-probability sample, inferential testing—including subgroup comparisons, chi-square analyses, logistic regression modelling, and adjusted confidence intervals—could not be conducted. Such analyses may yield unstable or misleading estimates under the current sampling structure; therefore, results were limited to descriptive statistics. Additionally, the composite knowledge score coded “Don’t know” responses as incorrect, following common KAP methodologies, although alternative scoring strategies (e.g., weighting domains or excluding policy-related items) may yield different point estimates.

These strengths and limitations should be considered when interpreting the findings and comparing them with studies from other regions.

### 4.2. Public Health and Policy Implications

Findings support the integration of G6PD-specific education into premarital and antenatal counselling, primary healthcare, and pharmacy-based advisory systems. The use of electronic medical alerts and expanded community awareness campaigns may help reduce preventable haemolytic crises, align with Saudi Vision 2030 health objectives, and strengthen hereditary disease prevention pathways.

These findings align with Vision 2030 preventive-health goals, particularly those emphasizing improved public health literacy, expanded access to preventive and premarital counselling services, and early identification of hereditary risks. Strengthening G6PD-related education within primary care, maternal and child health services, and digital-health platforms supports national objectives for enhanced community engagement and proactive disease prevention. Integrating targeted awareness initiatives into routine care pathways may help reduce preventable haemolytic crises and advance population-level genetic health outcomes.

## 5. Conclusions

This study offers the first community-level assessment of knowledge, attitudes, and practices regarding G6PD deficiency in Al-Kharj, revealing suboptimal overall knowledge and limited uptake of preventive behaviours. While common triggers (e.g., fava beans) and cardinal symptoms (e.g., jaundice) were relatively well recognised, misconceptions persisted around inheritance (X-linked), medication-induced haemolysis, and non-fava dietary items. Low engagement with premarital consultation and genetic assessment underscores gaps in prevention pathways.

Integrating targeted G6PD education into premarital, antenatal, and pharmacy-based services—supported by brief counselling scripts and medication safety prompts—could close critical knowledge gaps, promote early identification and counselling, and reduce preventable haemolysis. These actions are consistent with Saudi Vision 2030 priorities on preventive health and can help lessen the community burden of G6PD-related complications. Collaborative multi-sectoral research, including longitudinal and interventional designs, is recommended to evaluate the impact of targeted awareness programmes on future clinical and behavioural outcomes.

## Figures and Tables

**Table 1:** Sociodemographic characteristics of the participants (*N* = 1104).

Parameter	Category	*n*	%
Gender	Female	629	57.0
	Male	475	43.0
Age group	18–27 years	384	34.8
	28–37 years	392	35.5
	38–47 years	177	16.0
	48–57 years	66	6.0
	≥58 years	85	7.7
Education	Primary education	263	23.8
	Elementary education	287	26.0
	Secondary education	176	15.9
	University and above	378	34.2

**Table 2:** General awareness of G6PD deficiency among participants (*N* = 1104).

Parameter	Yes n (%)	No n (%)	Don’t Know n (%)
Heard of “fava bean anaemia”	645 (58.5)	249 (22.6)	210 (19.0)
G6PD deficiency is inherited	552 (50.0)	318 (28.8)	234 (21.2)
Sex is linked (X-linked)	420 (38.0)	208 (18.8)	476 (43.1)
Fava beans can trigger haemolysis	682 (61.8)	69 (6.2)	353 (32.0)
G6PD testing is part of premarital screening	334 (30.2)	65 (5.9)	705 (63.9)
Some medications can trigger haemolysis	399 (36.1)	270 (24.5)	435 (39.4)
Family history of G6PD deficiency	269 (24.4)	123 (11.1)	712 (64.5)
Know own G6PD status	263 (23.8)	416 (37.7)	425 (38.5)

**Table 3:** Knowledge of G6PD deficiency symptoms (*N* = 1104).

Symptom Item	Yes n (%)	No n (%)	Don’t Know n (%)
Pallor	401 (36.3)	275 (24.9)	428 (38.8)
Loss of appetite, nausea, diarrhoea, vomiting	389 (35.2)	83 (7.5)	632 (57.2)
Jaundice	598 (54.2)	82 (7.4)	424 (38.4)
Shortness of breath	388 (35.1)	278 (25.2)	438 (39.7)
Death or physical complications	380 (34.4)	295 (26.7)	429 (38.9)

**Table 4:** Knowledge of dietary and medication triggers (*N* = 1104).

Item	Yes n (%)	No n (%)	Don’t Know n (%)
Fava beans cause haemolysis	625 (56.6)	62 (5.6)	417 (37.8)
Hummus/chickpeas trigger haemolysis	565 (51.2)	106 (9.6)	433 (39.2)
Falafel (fava-based) causes haemolysis	598 (54.2)	77 (7.0)	429 (38.9)
Lentils trigger haemolysis	564 (51.1)	112 (10.1)	428 (38.8)
Blueberries trigger haemolysis	363 (32.9)	289 (26.2)	452 (41.0)
Nuts/peanuts trigger haemolysis	369 (33.4)	97 (8.8)	638 (57.8)
Soy products trigger haemolysis	364 (33.0)	96 (8.7)	644 (58.3)
Menthol-containing foods trigger haemolysis	378 (34.2)	277 (25.1)	449 (40.7)
Aspirin triggers haemolysis	370 (33.5)	282 (25.5)	452 (41.0)
Antimalarial drugs trigger haemolysis	367 (33.2)	274 (24.8)	463 (41.9)
Sulphonamide antibiotics trigger haemolysis	372 (33.7)	269 (24.4)	463 (41.9)
Smoking triggers haemolysis	560 (50.7)	82 (7.4)	462 (41.9)

**Table 5:** Preventive and screening practices related to G6PD deficiency (*N* = 1104).

Practice Item	Yes n (%)	No n (%)	Don’t Know n (%)
Medical consultation before marriage	458 (41.5)	230 (20.8)	416 (37.7)
Ever had a genetic assessment	238 (21.6)	459 (41.6)	407 (36.9)
Postnatal medical consultation to confirm status	229 (20.7)	464 (42.0)	411 (37.2)

**Table 6:** Attitudes toward G6PD deficiency (*N* = 1104).

Attitude Item	Yes n (%)	No n (%)	Don’t Know n (%)
G6PD deficiency is a serious problem	502 (45.5)	60 (5.4)	542 (49.1)
Consanguineous marriage contributes to G6PD deficiency	572 (51.8)	71 (6.4)	461 (41.8)
Pregnancy should be avoided if a family has an affected child	242 (21.9)	292 (26.4)	570 (51.6)
Patients should be monitored for life	502 (45.5)	48 (4.3)	554 (50.2)

## Data Availability

The data supporting the findings of this study are available from the corresponding author upon reasonable request.

## Supplementary Materials

The following supporting information can be downloaded at: https://www.mdpi.com/article/10.3390/healthcare13243261/s1, Supplementary File S1: Full bilingual questionnaire used in the study.
