# Supplementary material for: Public Knowledge, Attitudes, and Preventive Practices Toward G6PD Deficiency in Al-Kharj, Saudi Arabia: A Community-Based Cross-Sectional Study
Source: Healthcare (Basel). 2025 Dec 12;13(24):3261. doi: 10.3390/healthcare13243261 (PMC12732968; doi:10.3390/healthcare13243261)
Supplement: Supplementary file 1 [file healthcare-13-03261-s001.zip › healthcare-4025479-supplementary.pdf]

Supplementary File S1.

G6PD Knowledge, Attitudes, and Practices Questionnaire (English Version)

Section 1: Sociodemographic Characteristics of Participants:

1. **Age Group**
   - 18–27 years
   - 28–37 years
   - 38–47 years
   - 48–57 years
   - ≥ 58 years

2. **Gender**
   - Male
   - Female

3. **Education Level**
   - Primary education
   - Elementary education
   - Secondary education
   - University degree or higher

Section 2: Knowledge of G6PD Deficiency

Please answer each item with: Yes / No / Don't know

1. Heard of "fava-bean anaemia."
2. G6PD deficiency is an inherited condition.
3. G6PD deficiency is sex-linked (X-linked).
4. Eating fava beans can trigger haemolysis in people with G6PD deficiency.
5. G6PD is not part of the official premarital screening panel.
6. Some medications can trigger haemolysis in G6PD-deficient individuals.
7. I have a family history of G6PD deficiency.
8. I know my own G6PD status.

Section 3: Knowledge of Symptoms of G6PD Deficiency

Please answer each item with: Yes / No / Don't know

1. Pallor (pale skin)
2. Loss of appetite, nausea, diarrhoea, or vomiting

3. Jaundice (yellowing of the skin or eyes)
4. Shortness of breath
5. Severe complications such as death or long-term physical problems.

Section 4:Knowledge of Dietary and Medication Triggers of Haemolysis in G6PD Deficiency

Please answer each item with: Yes / No / Don't know

- **Foods and Dietary Triggers**

1. Eating Fava beans can trigger haemolysis.
2. Hummus/chickpeas can trigger haemolysis.
3. Falafel (made from fava beans) can cause haemolysis.
4. Lentils can trigger haemolysis.
5. Blueberries can trigger haemolysis.
6. Nuts or peanuts can trigger haemolysis.
7. Soy products can trigger haemolysis.
8. Menthol-containing foods or products can trigger haemolysis.

- **Medication Triggers**

9. Aspirin can trigger haemolysis in people with G6PD deficiency.
10. Antimalarial drugs can trigger haemolysis.
11. Sulphonamide antibiotics can trigger haemolysis.

- **Other Triggers**

12. Smoking can trigger haemolysis in people with G6PD deficiency.

Section 5:Screening and Preventive Practices

Please answer each item with: Yes / No / Don't know

1. I have sought medical consultation before marriage to assess genetic or hereditary risks.
2. I have undergone a genetic assessment or counselling related to hereditary conditions.
3. I have sought postnatal medical consultation to confirm my (or my child's) G6PD status.

Section 6:Attitudes Toward G6PD Deficiency

Please answer each item with: Yes / No / Don't know

1. G6PD deficiency is a serious problem.
2. Consanguineous marriage contributes to G6PD deficiency.
3. Pregnancy should be avoided if a family has an affected child.
4. Patients with G6PD deficiency should be monitored for life.
